# Identification and Molecular Characterization of Superoxide Dismutases Isolated From A Scuticociliate Parasite: Physiological Role in Oxidative Stress

**DOI:** 10.1038/s41598-019-49750-5

**Published:** 2019-09-16

**Authors:** Iria Folgueira, Jesús Lamas, Ana Paula de Felipe, Rosa Ana Sueiro, José Manuel Leiro

**Affiliations:** 1 Department of Microbiology and Parasitology, Laboratory of Parasitology, Institute of Research and Food Analysis, Campus Vida, University of Santiago de Compostela, E-15782 Santiago de Compostela, Spain; 2 Department of Fundamental Biology, Institute of Aquaculture, Campus Vida, University of Santiago de Compostela, E-15782 Santiago de Compostela, Spain

**Keywords:** Parasite physiology, Environmental impact

## Abstract

*Philasterides dicentrarchi* is a free-living microaerophilic scuticociliate that can become a facultative parasite and cause a serious parasitic disease in farmed fish. Both the free-living and parasitic forms of this scuticociliate are exposed to oxidative stress associated with environmental factors and the host immune system. The reactive oxygen species (ROS) generated by the host are neutralized by the ciliate by means of antioxidant defences. In this study we aimed to identify metalloenzymes with superoxide dismutase (SOD) activity capable of inactivating the superoxide anion (•O_2_^−^) generated during induction of oxidative stress. *P*. *dicentrarchi* possesses the three characteristic types of SOD isoenzymes in eukaryotes: copper/zinc-SOD, manganese-SOD and iron-SOD. The Cu/Zn-SOD isoenzymes comprise three types of homodimeric proteins (CSD1-3) of molecular weight (MW) 34–44 kDa and with very different AA sequences. All Cu/Zn-SODs are sensitive to NaCN, located in the cytosol and in the alveolar sacs, and one of them (CSD2) is extracellular. Mn- and Fe-SOD transcripts encode homodimeric proteins (MSD and FSD, respectively) in their native state: a) MSD (MW 50 kDa) is insensitive to H_2_O_2_ and NaN_3_ and is located in the mitochondria; and b) FSD (MW 60 kDa) is sensitive to H_2_O_2_, NaN_3_ and the polyphenol trans-resveratrol and is located extracellularly. Expression of SOD isoenzymes increases when •O_2_^−^ is induced by ultraviolet (UV) irradiation, and the increase is proportional to the dose of energy applied, indicating that these enzymes are actively involved in cellular protection against oxidative stress.

## Introduction

Scuticociliates are typically very abundant microphagous bacteriovores in lakes and coastal marine eutrophic habitats. They are usually associated with soft sediments accumulated on the bottom of these habitats and are very common in deep waters, in or below the oxycline, and are highly adapted to low levels of dissolved oxygen^1–6^. *Philasterides dicentrarchi* is a free-living scuticociliate that can transform into an opportunistic parasite^7^ and infect flatfish in culture, causing high mortality rates^8,9^. Like other microaerophilic ciliates, *P*. *dicentrarchi* can survive and remain viable under anoxic conditions or after cyanide treatment^10,11^. The microaerophilic condition of *P*. *dicentrarchi* will probably facilitate survival in the internal anoxic environment of the host, representing the first line of adaptation to parasitism in this ciliate^10^. In addition, we have observed that during the endoparasitic phase of infection, the cells of the innate immune system of turbot generate toxic products, including reactive oxygen species (ROS) such as superoxide (•O_2_^−^), hydrogen peroxide (H_2_O_2_) and hydroxyl radicals (•OH). The antioxidant cellular system limits the presence of ROS, preventing damage to macromolecules by these oxygen derivatives. This process involves several intracellular enzymes, such as superoxide dismutase (SOD), catalase (CAT), glutathione peroxidase (GPx) and peroxiredoxin (Prdx)^12–16^. These proteins are evolutionarily conserved in all eukaryotic organisms, ranging from yeast to higher organisms. Likewise, exposure of aquatic organisms, including ciliated protozoa, to thermal stress, ultraviolet radiation (UVR, 280–400 nm) or pollution can cause a significant increase in the cellular concentrations of ROS, which must be neutralized by detoxifying enzymes to prevent toxic effects^17,18^. It has been found that SOD activity increases after exposure of diatoms to variations in irradiance, including UVR, thereby reducing oxidative stress^19^. In eukaryotes, SOD enzymes are grouped into families based on the presence of different metal cofactors (Mn/Fe, Fe and Cu/Zn) at the active site of the enzyme in the protein fold and are located in different cell compartments^20^. Many eukaryotes, including several microaerobic or microaerophilic protists, have an extracellular SOD (EC-SOD or SOD3)^21,22^. More specifically, in ciliates such as *Euplotes*, *Tetrahymena* and *Strombidium*, several types of SODs in the Cu/Zn-SOD family have been identified and characterized, and the presence of Mn-SODs and Fe/Mn-SODs has been demonstrated^23–26^.

In the present study, we carried out biochemical and molecular characterization of enzymes with SOD activity present in the scuticociliate *P*. *dicentrarchi*, focusing on regulation of the expression of these enzymes under oxidative stress conditions.

## Materials and Methods

### Parasites

Specimens of *P*. *dicentrarchi* (isolate I1) were collected under aseptic conditions from peritoneal fluid obtained from experimentally infected turbot, *Scophthalmus maximus*, as previously described^27^. The ciliates were cultured at 21 °C in complete sterile L-15 medium, as previously described^28^. In order to maintain the virulence of the ciliates, the fish were experimentally infected every 6 months by intraperitoneal (ip) injection of 200 μL of sterile physiological saline containing 5 × 10^5^ trophozoites. The ciliates were then recovered from ascitic fluid and maintained in culture as described above.

### Experimental animals

Turbot of approximately 50 g body weight were obtained from a local fish farm. The fish were held in 250-L tanks with aerated recirculating sea water maintained at 14 °C. They were subjected to a photoperiod of 12 L:12D and fed daily with commercial pellets (Skretting, Burgos, Spain). The fish were acclimatized to laboratory conditions for 2 weeks before the start of the experiments.

Eight to 10-week-old Institute for Cancer Research (ICR) (Swiss) CD-1 mice, initially supplied by Charles River Laboratories (USA), were bred and maintained in the Central Animal Facility of the University of Santiago de Compostela (Spain). All experimental protocols carried out in the present study followed the European legislation (Directive 2010/63/EU) and the Spanish legislative requirements related to the use of animals for experimentation (RD 53/2013) and were approved by the Institutional Animal Care and Use Committee of the University of Santiago de Compostela (Spain).

### Purification of SODs by anion exchange chromatography

Ciliates were collected by centrifugation at 700 *g* for 5 min and resuspended in saline phosphate buffer (PBS) containing 1x protease inhibitor cocktail (Sigma-Aldrich). The ciliates present in the solution were then lysed by ultrasonic treatment (W-250 sonifier, Branson Ultrasonic Corporation, USA) and centrifuged at 15000 *g* for 20 min at 4 °C^29^. The supernatant thus obtained was dialyzed against a start buffer containing 20 mM Tris-HCl pH 8.0, before being filtered (0.45 μm) Samples of 1 mL of lysed extract of the ciliate (SE) were subjected to anion exchange chromatography (AEC). For this purpose, an AEC HiTrapQ column and an automatic protocol were integrated into the Äktaprime plus system (GE Healthcare, Sweden), and the sample was eluted using a buffer containing 20 mM Tris-HCl pH 8.0 and 1.0 M NaCl. The eluted sample was collected in 2 mL fractions. Those fractions associated with peaks determined by absorbance at 280 nm were pooled, dialyzed against distilled water, lyophilized and stored at −20 °C until analysis by native polyacrylamide gel electrophoresis, as described in detail below.

### Determination of SOD activity in native polyacrylamide gels

The SOD activity was determined on polyacrylamide gels (PAGE) following the method of Weydert and Cullen^30^. The ciliates were cultured at a concentration of 5 × 10^5^ trophozoites/mL in 24-well culture plates (Corning, USA) and were maintained under conditions of normoxia, with or without treatment with inhibitors: H_2_O_2_, KCN, NaN_3_ and *trans* resveratrol (RESV). After incubation for 30 min without or with the inhibitors (100 μM), the ciliate samples were collected by centrifugation at 700 *g* for 5 min and washed twice in incomplete L-15 medium (medium without bovine serum). The pellet containing the ciliates was then resuspended in a loading buffer containing 1.5 M Tris-HCl pH 6.8, 50% glycerol and 5% bromophenol blue, which lyses the ciliates by osmotic shock. In some experiments, lyophilized samples were separated by anion exchange chromatography and resuspended in loading buffer. The enzymatic activity of the samples was determined on native PAGE, formed by a 5% concentrating gel polyacrylamide in 1.5 M Tris-HCl buffer pH 6.8 and a 12.5% separating gel in Tris-HCl buffer pH 8.8. Gel polymerization was carried out by the addition of 0.04% ammonium persulphate (APS) and 0.0005% tetramethylethylenediamine (TEMED). After gel polymerization, a pre-electrophoresis step was carried out for 1 h at 20 mA in electrophoresis buffer containing 200 mM Tris-HCl pH 8.8 and 0.7 mM Na_2_EDTA at 4 °C to remove the remains of any APS, which can inactivate the enzyme. All of the initial buffer was then removed, and the samples were prepared in loading buffer and placed in the concentrator gel. Electrophoresis was finally carried out in an electrophoresis buffer containing 50 mM Tris-HCl pH 8.3, 1.5 mM Na_2_EDTA and 0.3 M glycine, for 1.5 h at 50 mA. The electrophoresed gels were washed twice in distilled water and stained with a solution of nitroblue tetrazolium chloride (NBT) (2.43 M NBT, 28 mM TEMED, 0.14 M riboflavin-5′-phosphate) for 20 min, with agitation at room temperature. The gels were then washed twice with distilled water and exposed, while still in the distilled water, to light for 12 h. The presence of enzymatic activity was observed by the appearance of destained bands on the violet-stained gel.

### RNA-Seq

For analysis of the ciliate transcriptome, trophozoites (10^7^) were concentrated by centrifugation, frozen in liquid nitrogen and sent on dry ice to Future Genomic Technologies (Leiden, Netherlands) for RNAseq analysis. Transcript sequences from Illumina RNA-Seq data (fragments of approximately 100 pb), obtained by amplification by SBS, were assembled using Trinity software (v2.6.5)^31^, included in the Galaxy application (https://usegalaxy.org/). The assembled sequences were analyzed by homology modelling, with Blastgo 5.0 software (Biobam, Spain), and annotated. Those sequences that encode proteins that are potentially related to the ciliate SODs were then selected from the *Tetrahymena thermophila* gene and protein sequences database using the BLASTx tool Wiki TGD (http://www.ciliate.org/blast/blast_link.cgi).

### Production of recombinant proteins in yeast cells

The complete nucleotide sequence that encodes the SODs (Pd-Cu/Zn-SOD2, Pd-Cu/Zn-SOD3, Pd-Fe-SOD and Pd-Mn-SOD) was obtained using an open reading frame search tool (ORF Finder; https://www.ncbi.nlm.nih.gov/orffinder/), from the annotation data obtained by analysis of the RNA-Seq. The nucleotide sequence was modified and optimized to produce a recombinant protein in the yeast *Klyuveromyces lactis* by using Integrated DNA Technologies (IDT) bioinformatics tool (https://eu.idtdna.com/CodonOpt). The gene was synthetized by Invitro GeneArt Gene Synthesis (ThermoFisher Scientific). The *K*. *lactis* Protein Expression kit (New England Biolabs, UK) and the pKLAC2 vector were used following the instructions provided by the kit, along with the protein secretion strategy, to express the recombinant protein in yeast. The synthesized nucleotide sequence was initially cloned in the pSpark^®^ II vector (Canvax, Spain), and recombinant plasmid was subsequently amplified in competent *Escherichia coli* strain DH-5α. After extraction and purification of the bacterial plasmid, a PCR was carried out with the following primers: Pd-Cu/Zn-SOD2 (FCSD2/RCSD2 primers): 5′-CGCCTCGAGAAAAGAATGTTGTTCGTCTTTCAGCGT-3′/5′ ATAAGAATGCGGCCGCTTAATGGTGATGATGGTGGTGGTG-3′; Pd-Cu/Zn-SOD3 (FCSD3/RCSD3 primers): 5-CGCCTCGAGAAAAGAATGCATGCCATTTGTATA-3′/5′-ATAAGAATGCGGCCGCTTAATGATGATGGTGGTG3′; Pd-Fe-SOD (FFSD/RFSD primers): 5′-CGC CTC GAG AAA AGA ATG AAC AAG TACATAATA-3′/5′-ATAAGAATGCGGCCGCTTAATGGTGATGGTGATGATG3′; and Pd-Mn-SOD (FMFSD/RMFSD primers): 5′-CGC CTC GAG AAA AGAATGAAATCGTTGACCAAA-3′/5′-ATAAGAATGCGGCCGCTTAATGATGGTGGTGATGGTG-3′. The nucleotide sequence that encodes the recombinant proteins included 10 codons encoding histidines at the C-terminal end of the protein. The yeasts were then transformed with the cloned pKLAC2 plasmid, grown for 2 h at 30 °C in YPGlu medium and seeded in YCB agar medium plates containing 5 mM acetamide at 30 °C for 3–4 days until formation of colonies. Several colonies were inoculated into the YPGal medium at 30 °C for 3–4 days in agitation at 250 rpm. Once an acceptable cell density was reached, the medium was centrifuged at 6000 g for 10 min, and the supernatant was maintained at 4 °C until use. The recombinant proteins [Pd-Cu/Zn-SOD (rCSD2), Pd-Cu/Zn-SOD3 (rCSD3), Pd-Fe-SOD (rFSD) and Pd-Mn-SOD (rMSD)] were purified by affinity chromatography, using prepacked columns with Ni-Sepharose (HisTrap^TM^, GE Healthcare) in an ÄKTA Star protein purification system (GE Healthcare), following the manufacturer′s instructions. After elution, the protein was dialysed against distilled water in a dialysis tube of 3 kDa pore size. Finally, the protein was lyophilized and kept at 4 °C until use.

### Transmission electron microscopy (TEM)

For TEM analysis we followed the technique described by Paramá *et al*.^32^. Briefly, the cultured ciliates were collected by centrifugation at 1000 g for 5 min. Cells were fixed in 2.5% (v/v) glutaraldehyde in 0.1 M cacodylate buffer at pH 7.2. They were then washed several times with 0.1 M cacodylate buffer and post-fixed in 1% (w/v) OsO_4_, pre-stained in saturated aqueous uranyl acetate, dehydrated through a graded acetone series and embedded in Spurr’s resin. Semi-thin sections were then cut with an ultratome (Leica Ultracut UCT, Leica microsystems, Germany) and stained with 1% toluidine blue for examination by light microscopy. Ultrathin sections were stained in alcoholic uranyl acetate and lead citrate and viewed in a Jeol JEM-1011 transmission electron microscope (Jeol, Japan) at an accelerating voltage of 100 kV.

### Exposure of the ciliates to ultraviolet radiation

Ciliates (5 × 10^5^ ciliates mL^−1^) were cultured in 12-well culture plates (Corning, USA) in a final volume of 2 mL of complete L-15 medium. The plates were inserted in a UV crosslinker (UVC500, Hoefer, USA) until reaching energies of 1, 2 and 3 Joules/cm^2^ (J/cm^2^). At the end of the exposure period, the viability of the ciliates was checked by observing their morphology and motility under an inverted microscope. The ciliates were centrifuged at 700 g for 5 min, the supernatant was removed, and the pellet was frozen at −20 °C until use.

### Immunizations and serum collection

A group of five ICR (Swiss) CD-1 mice were immunized by ip injection with 200 μL per mouse of a solution of 200 μg of recombinant proteins (rCSD2,3, rFSD and rMSOD) in 1% chitosan hydrogel (CH), prepared according to the method of Barua and Das^33^. The mice were injected ip with the same dose of purified recombinant proteins in CH, 15 and 30 days after first immunization. Each mouse was bled via retrobulbar venous plexus 7 days after the final injection, and if the antibody level was satisfactory, the mouse was completely bled by decapitation. The blood was left to coagulate overnight at 4 °C, and the serum was separated by centrifugation at 2000 × g for 10 min, mixed 1:1 with glycerol and stored at −20 °C until use.

### Sodium dodecyl sulphate polyacrylamide gel electrophoresis (SDS-PAGE) and Western-blot

**Figure 1:**
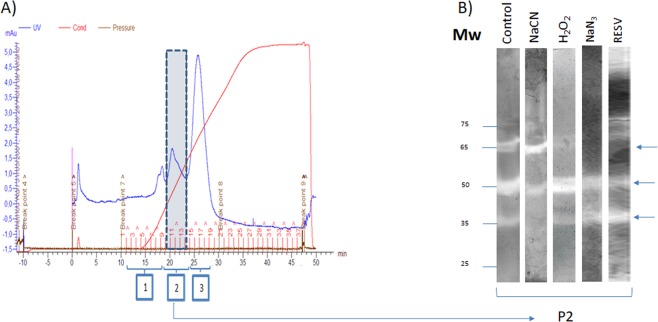
(**A**) Profile of a *P*. *dicentrarchi* lysate eluted through an anion exchange column (HiTrap Q) in ÄKTAprime plus (GE Healthcare) equipment. After chromatography, the fractions corresponding to the three major peaks obtained after separation of the sample (1–3) were subjected to native polyacrylamide gel electrophoresis (PAGE) staining to analyse the enzymatic activity. The enzymatic activity SOD (arrows) was located mostly in a peak (P2). (**B**) SOD activity observed in 12.5% native polyacrylamide gel electrophoresis (PAGE) visualized by the nitroblue tetrazolium reduction assay. In the assay, purified protein fractions, obtained by ion exchange chromatography (P2), were incubated with sodium cyanide (NaCN), hydrogen peroxide (H_2_O_2_), sodium azide (NaN_3_) and resveratrol (RESV), or without inhibitors (control). The arrows indicate the bands of SOD activity. Mw (molecular weight).

SDS-PAGE of the recombinant SODs (rCSD3, rFSD and rMSD) and peak 2 (P2) obtained by AEC (see Fig. 1) from *P*. *dicentrarchi* not exposed or exposed to different levels of UV radiation was performed on linear 12.5% polyacrylamide mini gels in a Mini-Protean^®^ Tetra cell system (BioRad, USA), as described by Iglesias *et al*.^34^. Samples were included in a loading buffer with 62 mM Tris-HCl buffer, pH 6.8, containing 2% SDS and 10% glycerol. The gels consisted of 4% stacking gel and 12.5% linear separating gel. Samples were dissolved in 62 mM Tris-HCl buffer pH 6.8 with 2% SDS, 10% glycerol and 0.004% bromophenol blue, and they were then heated for 5 min in a boiling water bath. Electrophoresis was carried out at a constant 200 V in Tris-glycine electrode buffer (25 mM Tris, 190 mM glycine, pH 8.3).

For Western-blot analysis, samples separated by electrophoresis were immunoblotted at 15 V for 35 min to Immobilon-P transfer membranes (0.45 μm; Millipore, USA) in a trans-blot SD transfer cell (Bio-Rad, USA) with electrode buffer containing 48 mM Tris, 29 mM glycine, 0.037% SDS and 20% methanol, pH 9.2. Membranes were washed with Tris buffer saline (TBS; 50 mM Tris, 0.15 M NaCl, pH 7.4) and stained with Ponceau S to verify transfer, blocked for 2 h at room temperature with TBS containing 0.2% Tween 20 and 5% non-fat dry milk, before being washed in TBS and incubated for 1 h with sera of immunized mice anti-rCSD2–3, anti-rFSD and anti-rMSD (1:100 dilution). All samples were then incubated with peroxidase-conjugated rabbit anti-mouse Ig (Dakopatts; dilution 1:800) and finally with 0.003% H_2_O_2_ and 0.06% 3,30-diaminobenzidine tetrahydrochloride containing 0.03% NiCl_2_ (DAB/NiCl_2_, Sigma, USA). The reaction was stopped after approximately 3 min by exhaustive washing with TBS. Membranes were scanned, and the bands obtained were quantified on the basis of their signal intensity in the images by using *Image J* software (https://imagej.nih.gov/ij/).

### Immunofluorescence and confocal microscopy

For immunolocalization of Pd-Cu/Zn-SOD3 and Pd-Mn-SOD in the trophonts, an immunofluorescence assay was performed, as previously described^35^. Briefly, ciliates (5 × 10^6^) were centrifuged at 700 × g for 5 min, washed twice with PBS pH 7.0 and fixed for 15 min in a solution of 4% formaldehyde in PBS at room temperature. The ciliates were then washed twice with PBS, resuspended in a solution containing 0.3% Triton X-100 in PBS for 3 min, washed twice with PBS and incubated with 1% BSA for 30 min. After this blocking step, the ciliates were washed in PBS and incubated at room temperature with stirring at 750 rpm for one hour with a 1:100 dilution in PBS of mouse sera anti-rCSD3 and anti-rMSD. The samples were washed 3 times with PBS, before fluorescein isothiocyanate (FITC) conjugated rabbit/anti-mouse Ig (DAKO, Denmark) (dilution, 1:1000) was added, and the samples were incubated for 1 h at room temperature and in darkness. After another three washes in PBS, the samples were mounted in PBS-glycerol (1:1) and visualized by confocal microscopy (Leica TCS-SP2, Leica Microsystems, Germany).

### Phenazine methosulfonate (PMS)-nitroblue tetrazolium (NBT) assay

SOD activity was measured spectrophotometrically in the PMS-NBT assay^36^. In this assay, reduction of NBT occurs by •O_2_^−^ generated by a mixture of nicotinamide adenine dinucleotide (NADH) and PMS at non-acidic pH. This was done by adding 100 μL of 1 mg/mL of the P2 of *P*. *dicentrarchi*, or 100 μL of P2 from ciliates treated with UV at 3 J/cm^2^, containing 50 μM of NBT and 78 μM NADH in 100 mM sodium phosphate buffer (PB) at pH 7.4, to each well of 96-well microplates (Corning, USA). The reaction was started by adding 100 μL of PMS (5 μM PMS in 100 mM PB pH 7.4). Assay mixtures were incubated at 25 °C for 60 min. The blue formazan resulting from the reduction of NBT by •O_2_^−^ generated from autooxidation of PMS was measured spectrophotometrically at 560 nm. As the enzymatic activation proceeds, a reduction in the blue colour generated is produced. The enzymatic activity was quantified as the decrease in absorbance at 560 nm/min.

### Enzyme-linked immunosorbent assay (ELISA)

The CSD2, CSD3, FSD and MFSD proteins in the culture medium and in the trophonts after exposure to ultraviolet radiation at 3 J/cm^2^ were detected and quantified by ELISA. Briefly, 1 µg of P2 peak from AEC of ciliates exposed or not exposed to UV radiation or purified recombinant SODs (rCSD2, rCSD3, rFSD and rMSD) in 100 µl of carbonate-bicarbonate buffer pH 9.6 (coupling buffer), or 90 μL of incomplete L-15 medium from ciliates exposed or not exposed to UV radiation and 10 μL of coupling buffer 10×, was added to 96-well hydrophilic, protein-binding plates (ThermoFisher Scientific, USA) and incubated overnight at 4 °C. The plates were then washed 3 times with TBS (50 mM Tris, 0.15 M NaCl, pH 7.4), blocked for 1 h with TBS containing 0.2% Tween 20 (TBS-T_1_), 5% non-fat dry milk, incubated for 15 min at 37 °C and at 750 rpm in a microplate shaker with 100 µl of a 1:100 dilution (in TBS-T_1_ containing 1% non-fat dry milk) of immunized mice serum (anti-rCSD2, anti-rCSD3, anti-rFSD and anti-rMSD, serum), and washed 5 times with TBS containing 0.05% Tween 20. Bound mouse antibodies were detected with peroxidase-conjugated rabbit anti-mouse Ig (Dako) diluted 1:1000 in TBS-T_1_ and incubated for 15 min at 37 °C and 750 rpm. The plates were then washed 5 times in TBS, and 100 µl of 0.04% *o-*phenylendiamine (OPD; Sigma) prepared in phosphate-citrate buffer, pH 5.0, containing 0.001% H_2_O_2_ was added to each well. The reaction was stopped after 20 min, with 3 N H_2_SO_4_, and the optical density (OD) was measured at 492 nm in an ELISA reader (Titertek Multiscan, Flow Laboratories).

### Reverse transcriptase-quantitative polymerase chain reaction (RT-qPCR)

Total RNA of 10^6^ trophozoites/mL of *P*. *dicentrarchi* not exposed or exposed to ultraviolet radiation was isolated with a NucleoSpin RNA isolation kit (Macherey-Nagel), following the manufacturer’s instructions. After purification of the RNA, the quality, purity and concentration were measured in a NanoDrop ND-1000 Spectrophotometer (NanoDrop Technologies, USA). The reaction mixture (25 μL) used for cDNA synthesis contained 1·25 μM random hexamer primers (Promega), 250 μM of each deoxynucleoside triphosphate (dNTP), 10 mM dithiothreitol (DTT), 20 U of RNase inhibitor, 2·5 mM MgCl_2_, 200 U of Moloney murine leukemia virus reverse transcriptase (MMLV; Promega) in 30 mM Tris and 20 mM KCl (pH 8·3) and 2 μg of sample RNA. PCR (for cDNA amplification) was performed with gene-specific primers forward/reverse pair for the Pd-Cu/Zn-SOD3 gene (FSD3/RSD3 primers): 5′-CAAAACCGCAGGTTCTCATT-3′/5′-CTTCTTGGGCATGAACCACT-3′. In parallel, a qPCR was performed with *P*. *dicentrarchi* elongation factor 1-alpha gene (EF-1α) (GenBank accession KF952262) as a reference gene, by including the forward/reverse primer pair (FEF1A/REF1A) 5′-TCGCTCCTTCTTGCATCGTT-3′/5′- TCTGGCTGGGTCGTTTTTGT-3′. The Primer 3Plus program was used, with default parameters, to design and optimize the primer sets. The PCR mixtures (25 μL) contained PCR reaction buffer (10 mM Tris-HCl, 50 mM KCl, 1·5 mM MgCl2, pH 9·0), 0·2 mM of each deoxynucleoside triphosphate (dNTPs, Roche), 0·4 μM of each primer, 3 units of recombinant Taq polymerase (NZY Taq DNA polymerase, NZYTech, Portugal) and 2 μL of cDNA. Quantitative PCR mixtures (10 μL) contained 5 μL Kapa SYBR FAST qPCR Master Mix (2×) (Sigma-Aldrich), 300 nM of the primer pair, 1 μL of cDNA and RNase-DNase-free water. Quantitative PCR was run at 95 °C for 5 min, followed by 40 cycles at 95 °C for 10 s and 60 °C for 30 s, ending with melting-curve analysis at 95 °C for 15 s, 55 °C for 15 s and 95 °C for 15 s. qPCRs were performed in an Eco RT-PCR system (Illumina). Relative quantification of gene expression was determined by the 2^−ΔΔCt^ method applied with software conforming to minimum information for publication of RT-qPCR experiments (MIQE) guidelines^37^.

### Bioinformatic and statistical analysis

InterPro software^38^ was used for functional analysis of proteins and classification into different families predicting the domains and important sites. The Phobius^39^, SignalP^40^ and Signal-3L ver. 2.0^41^ programs were used to predict the topology of transmembrane and location of signal peptide cleavage sites in AA (AA) sequences. The MitoProt II-v1.101 program^42^ was used to analyze the N-terminal region of the protein that might contain a mitochondrial signal sequence and its cleavage site. The MotiFinder tool of the Japanese GenomeNet network, accessible online at https://www.genome.jp/tools/motif/MOTIF.html was used to search for protein sequence motifs. The ProtParam tool was used to predict the physicochemical parameters for a given protein^43^. The SWISS-MODEL Protein server was used for modelling^40^. The bioinformatic tools LocTree3^42^ and PredictProtein^43^ were used to predict sub-cellular localization. The Clustal Omega multiple sequence alignment program was used to align the aa sequences of Pd-Cu/ZnSOD3 and Pd-Fe-SOD^44^. The Maximum Likelihood (ML) trees were constructed with a method based on a JTT model^45^ with the Mega7 program^46^, and the reliability of internal branches was assessed using a nonparametric bootstrap method with 1,000 replicates. The Bayesian inference (BI) analysis was performed with MrBayes 3.2.6^47^.

The values shown in the text and figures are means ± SEM. One-way analysis of variance (ANOVA) was used for comparison of more than two samples, and the Tukey-Kramer test was used for pairwise comparisons. The Student’s t-test was used for comparison of two samples. In both cases, differences were considered significant at *P* < 0.05.

## Results

### Isolation of the SODs and evaluation of enzymatic activities

For identification and purification of *P*. *dicentrarch*i SODs, a soluble extract of the ciliate (SE) was separated by anion exchange chromatography (AEC). The fractions thus obtained were analyzed by native electrophoresis, to detect enzymatic activity.

The results presented in Fig. 1A show the chromatographic profile obtained after separation of the SE fractions obtained by AEC by elution of the sample with the elution buffer. Three peaks of maximum absorption at 280 nm were observed. Analysis of the SOD activity of the three fractions, by non-denaturing PAGE, shows that the enzymatic activity occurs exclusively in the second elution peak (P2), detecting the appearance of three major bands with relative molecular weights of approximately 35, 50 and 60 kDa respectively (Fig. 1B).

Addition of inhibitors of SOD activity, such as sodium cyanide, hydrogen peroxide, sodium azide and the polyphenol resveratrol (RESV), at a concentration of 100 μM, caused inhibition of SOD activity (Fig. 1B). Specifically, NaCN caused total inhibition of the activity of SODs of mw 35 kDa; H_2_O_2_ caused total inhibition of the enzymatic activity of SODs of mw 65 kDa; while both NaN_3_ and RESV produced complete inhibition of the activity of SOD enzymes of mw 65 kDa (Fig. 1B).

### Molecular characterization of the SOD enzymes of *P. dicentrarchi*

**Figure 2:**
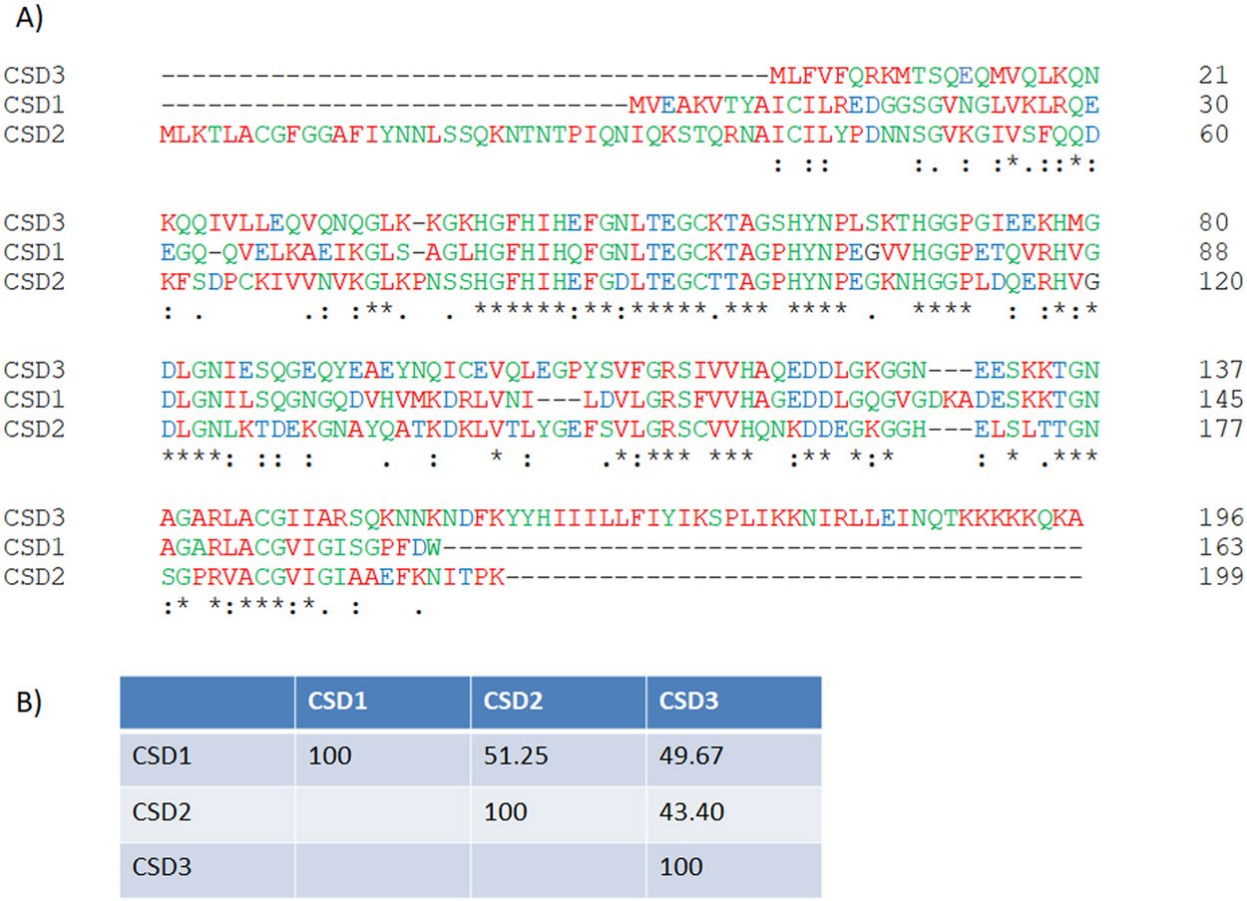
(**A**) Clustal Omega alignments of aa sequences of three Cu/Zn-SODs (CSD1-3) proteins found in *P*. *dicentrarchi* (strain I1). The symbols at the bottom indicate the conservation levels of the sequences: (*) fully conserved; (:) highly conserved; (.) conserved substitution. (**B**) Percent Identity Matrix of the sequences of the three proteins created by Clustal Omega 2.1.

After annotation of the transcripts obtained in an RNAseq experiment, we found that *P*. *dicentrachi* expressed three transcripts that encode proteins homologous to the Cu/Zn-SOD enzymes of ciliates (Fig. 2A), a transcript that encodes a protein homologous to Mn/Fe-SOD and a transcript that encodes a protein homologous to Fe-SOD. In homology modelling of the structure of the proteins encoded by the transcripts, the DeepView program (Swiss Pbd-Viewer) predicted that the oligomeric state of the proteins is dimeric.

Figure 2A shows the aa sequences of three transcripts obtained from an RNAseq assay. Analysis by the InterPro bioinformatics tool, designed for the analysis and classification of proteins, predicted that the aa sequences belong to the Cu/Zn-SOD superfamily. Regarding the biochemical parameters, the transcript corresponding to the Pd-Cu/Zn-SOD1 form has an ORF of 492 bp (GenBank accession number MH427342), which encodes a protein (CSD1) of 163 AA, with an estimated mw of 17173.30 Da and an estimated isoelectric point (pI) of 5.70. The transcript corresponding to the Pd-Cu/Zn-SOD2 form has a 600 bp ORF (GenBank accession number MK348945) that encodes a protein (CSD2) of 199 AA, with an estimated mw of 21385.98 daltons and an estimated pI of 8.18. The transcript related to the Pd-Cu/Zn-SOD3 form possesses an ORF of 591 bp (GenBank accession number MK348946) that encodes a protein (CSD3) of 196 AA, with estimated MW of 22197.45 Da and an estimated pI of 9.37. Global alignment of the aa sequences of the 3 proteins related to the Pd-Cu/Zn-SODs generated some regions with conserved domains (Fig. 2A); however, the global identity of the aa sequences is low (43–51%) (Fig. 2B). The gene ontology annotations of these sequences indicate that their molecular function is related to metal ion binding and biological process of oxidation-reduction and superoxide metabolic process. According to Signal-3L 2.0, a bioinformatic program predictor of signal peptides, CSD2, has a signal peptide between AA_1–40_. However, the Phobius bioinformatic predictor of signal peptides and transmembrane topology, predicted a signal peptide in the CSD2 protein between AA_1–20_. The topology of the CSD3 protein predicted by the Phobius bioinformatics tool indicates a cytoplasmic region between the AA_1–157_, a transmembrane region located between the AA_158–176_ and a non-cytoplasmic region between the AA_177–196_.

We also found that *P*. *dicentrarchi* has two SODs dependent on Fe and Mn/Fe, as a result of the analysis of *P*. *dicentrarch*i sequences with the Blastx program and using the *Tetrahymena thermophila* (AA) database for the search. The Mn-dependent enzyme (Pd-Mn-SOD) has a 663 bp ORF (GenBank accession number MK348948) that encodes a protein (MSD) of 220 AA, of MW of about 25074.43 Da and with an estimated pI of 7.06. This protein contains an export signal to the mitochondria between AA_1–12_ with a cleavage site al AA_13_. The Pd-Fe-SOD enzyme has a 750 bp ORF (GenBank accession MK348949) that encodes a protein (FSD) of 249 AA with an estimated MW of 29154.81 Da and an estimated pI of 5.79. The Phobius tool predicts for the FSD protein a signal peptide between the AA_1–21_. The homology modelling of the structure of the proteins encoded by the transcripts corresponding to the Pd-Mn- and Fe-SOD forms revealed the existence of an oligomeric state of the protein forming a homodimer.

**Figure 3:**
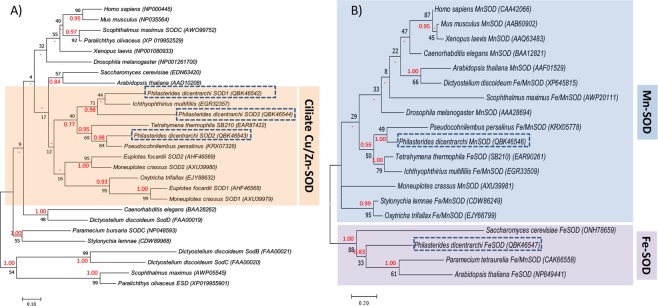
Phylogenetic analysis of (**A**) *P*. *dicentrarchi* Cu/Zn-SODs and (**B**) Fe/Mn-SOD proteins by the Maximum Likelihood (ML) and Bayesian inference (BI) methods, respectively. The trees with the highest log likelihood (**A**) (−4402.19) and (**B**) (−1780.26) are shown. Evolutionary analysis was conducted with the MEGA7 and MrBayes 3.2.6. programs. Initial trees for the heuristic search were obtained automatically by applying Neighbor-Joining and BioNJ algorithms to a matrix of pairwise distances estimated using a JTT model, and then selecting the topology with superior log likelihood value. The analysis involved (**A**) 27 and (**B**) 19 AA sequences. Numbers on branches represent the bootstrap value for ML analysis (black) and posterior probability value of BI analysis (red). The codes that appear next to the species in parentheses are the GenBank sequence accession numbers. Dashes (−) indicate disagreement between the ML and BI analysis. The scale bar corresponds to 10 (**A**) or 20 (**B**) substitutions per 100 AA positions.

The molecular evolution of Pd-Cu/Zn-SODs and Pd-Mn/Fe-SODs enzymes was studied within the framework of known SODs sequences of ciliate species and other species belonging to the most representative eukaryote groups (Fig. 3). The phylogenetic tree obtained by maximum likelihood (ML) and Bayesian inference (BI) methods indicate that Cu/Zn-SODs of *P*. *dicentrarchi* are closely related to Cu/Zn-SODs of ciliates and more separated from the SODs of other species of eukaryotes (Fig. 3A). More specifically, the Pd-Cu/Zn-SOD1–3 are closely related to a Cu/Zn-SOD of the ciliate *Ichthyophthirius multifiliis*, while the Pd-Cu/Zn-SOD2 is more closely related to a SOD of the scuticociliate *Pseudocohnilembus persalinus* (Fig. 3A). With respect to the Pd-Mn/Fe-SODs isoenzymes, the phylogenetic analysis confirms that both enzymes are related to the SODs of the Mn-SOD and Fe-SOD types of eukaryotes (Fig. 3B). On the other hand, the phylogenetic tree shows that Pd-Mn-SOD is grouped with Fe/Mn-SODs of ciliates and closely related to a Fe/Mn-SOD of the scuticociliate *P*. *persalinus*, while the Pd-Fe-SOD is grouped with a Fe/Mn-SOD from the ciliate *Paramecium* and a Fe-SOD from the plant *Arabidopsis* (Fig. 3B).

### Cellular localization of SODs in *P. dicentrarchi*

**Figure 4:**
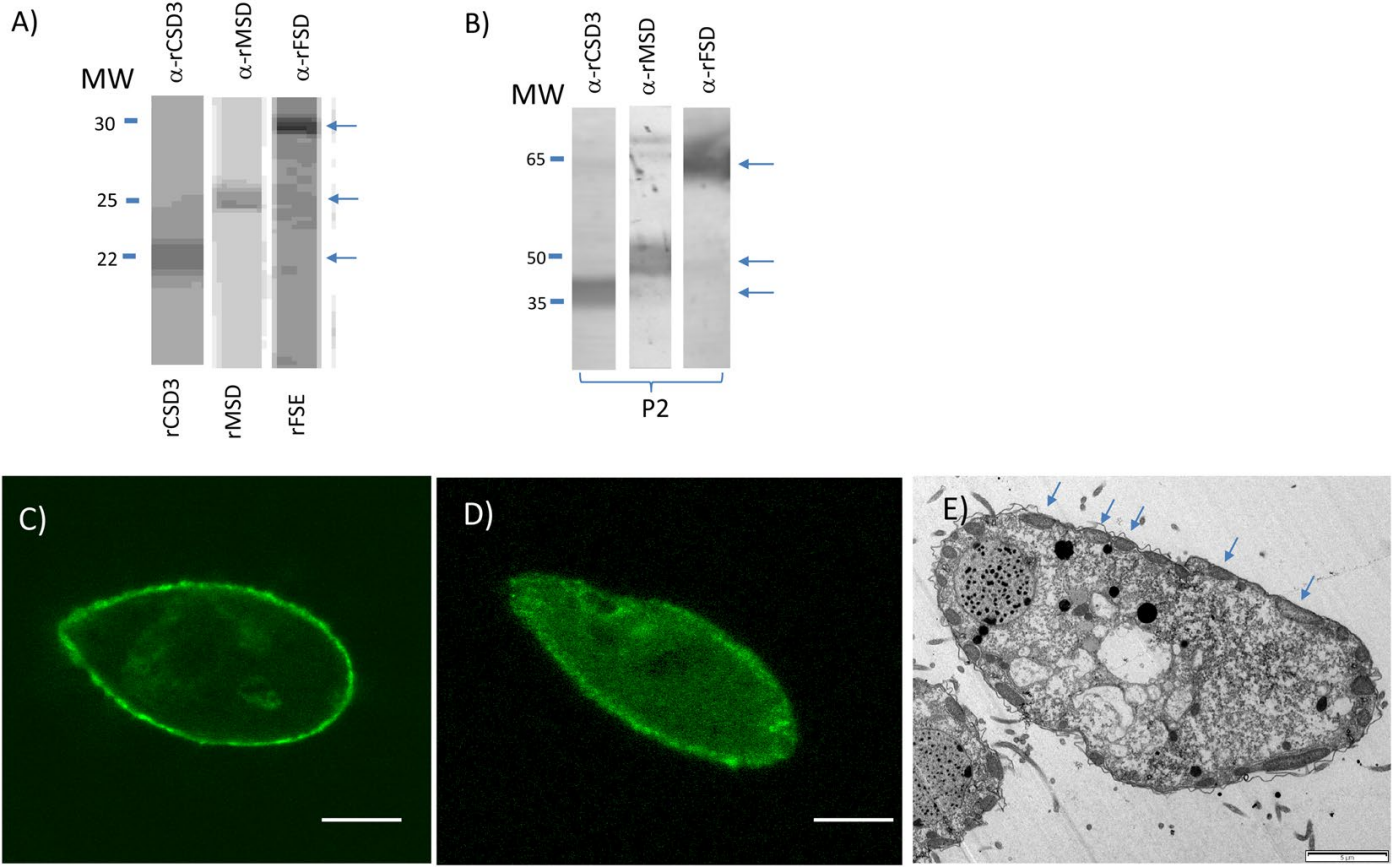
Western-blot analysis with polyclonal antibodies against the monomers of the recombinant proteins of the form of the Cu/Zn-SOD3 isoenzyme (rCSD3), the Mn-SOD isoenzyme (rMSD) and the Fe-SOD isoenzyme (rFSD) produced in (**A**) the yeast *Klyveromyces lactis*, and (**B**) against fractions purified by ion exchange chromatography with SOD activity (peak 2, P2) under non-reducing conditions, with the same antibodies as in A. molecular weight (Mw) markers are shown in kDa. Immunostaining patterns by confocal microscopy with (**C**) the polyclonal anti-CSD3 and (**D**) the anti-MSD antibodies. (**E**) Transmission electron micrograph of a *P*. *dicentrarchi* trophont showing the presence of aligned mitochondria (arrows), below the alveolar sacs. Bar size: 10 μm.

We used two approaches to determine the subcellular localization of the Pd-SOD isoenzymes: (1) bioinformatic prediction and (2) analysis by indirect immunofluorescence assay. In the first case, we used several bioinformatics tools such as PredictProtein and LocTree3 and, in the second case, we generated antibodies against CSD3 and MSD proteins (Fig. 4). Bioinformatic predictions indicate that the enzymes Pd-Cu/Zn-SOD1 and Pd-Cu/Zn-SOD2 would be located in the cytosol, while the Pd-Cu/Zn-SOD3 isoenzyme would be located in structures related to plant chloroplasts or in the periplasmic space of bacteria. The subcellular localization of Pd-Mn-SOD is predicted to be in the mitochondria and the Pd-Fe-SOD in the cytoplasm. In the second case, in order to determine the cellular localization of the different Pd-SODs by immunofluorescence analysis, we generated antibodies against the recombinant proteins corresponding to Pd-Cu/Zn-SOD3 (rCDS3) and Pd-Mn-SOD (rMSD) (Fig. 4A). The size of the recombinant monomeric proteins corresponds to the original ciliated proteins from which they were derived: 21 kDa for the CSD3 protein monomer, 25 kDa for the MSD protein monomer and between 29–30 kDa for the FSD protein monomer (Fig. 4A). In a Western blot, the antibodies generated against the recombinant monomeric proteins recognize native proteins of the ciliate bands of twice the molecular weight of the monomers (Fig. 4B). After mice were immunized with the different recombinant proteins, the antibodies obtained were tested against *P*. *dicentrarchi* trophozoites by immunofluorescence analysis, which revealed that the anti-rCSD3 antibodies recognised the alveolar sacs (Fig. 4C). The antibodies generated by injection with the rMSD recombinant protein strongly recognised the mitochondria (Fig. 4D) aligned just below the plasma membrane of the trophozoite (Fig. 4E).

### Expression of SODs in trophozoites of *P. dicentrarchi* exposed to ultraviolet radiation and oxidative stress

**Figure 5:**
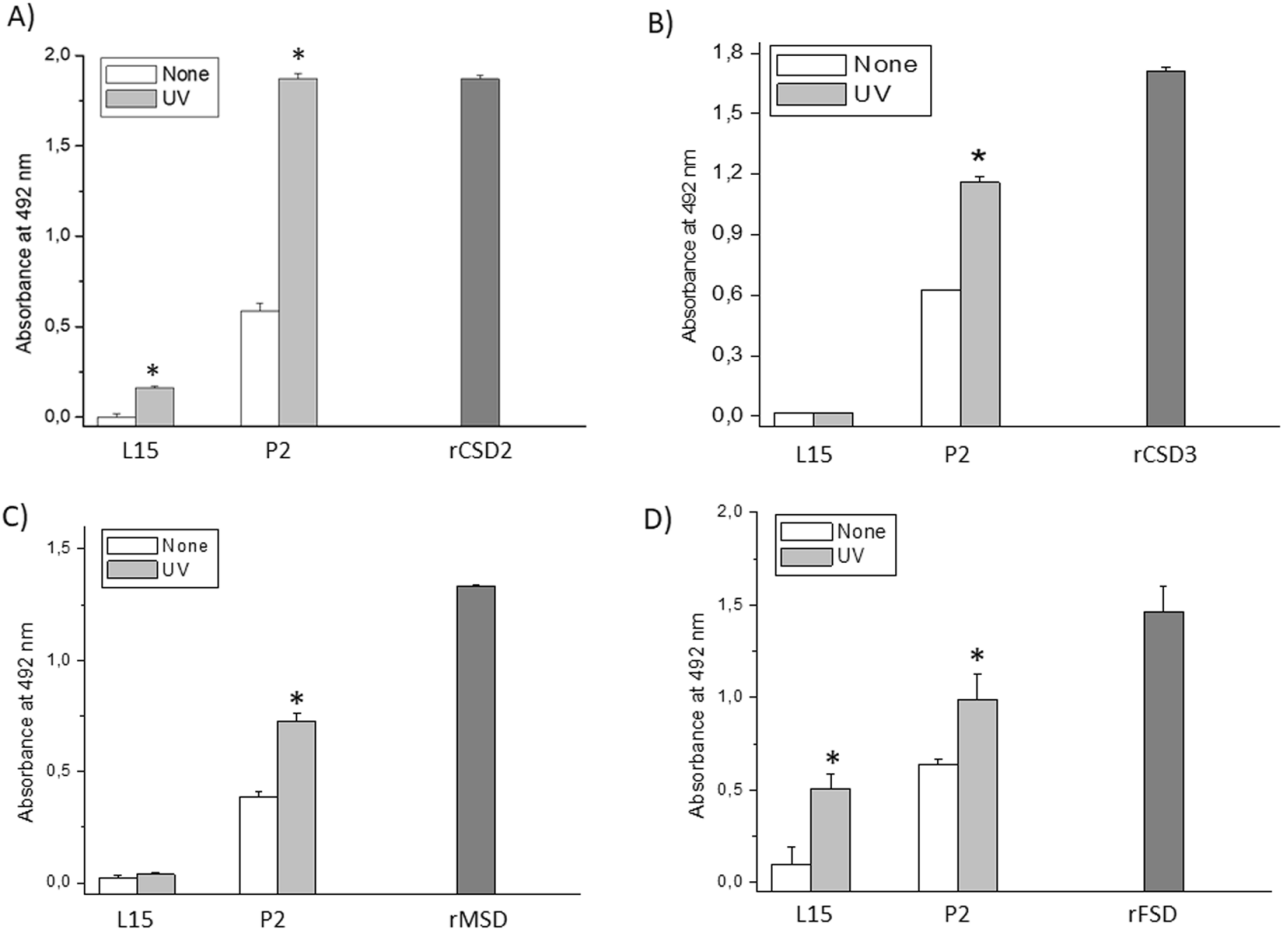
ELISA assay for the determination of expression levels of SODs in *P*. *dicentrarchi* trophonts exposed *in vitro* to ultraviolet (UV) radiation (3 J) relative to non-radiated trophonts (none). (**A**,**B**) The assay included polyclonal antibodies against the recombinant proteins of forms 2 and 3 of Cu/ZnSOD (anti-rCSD2 and anti-rCSD3, respectively), (**C**) Mn-SOD (anti-rMSD) and (**D**) Fe-SOD (anti-rFSD). The level of recognition was evaluated against the SODs present in peak 2 (P2) purified by ion exchange chromatography (see Fig. 1), using the proteins recombinants (rCSD2, rCSD3, rMSD and rFSD) as response controls and a sample of L5 culture medium to determine the presence of extracellular forms of SOD. The asterisks indicate statistically significant differences (*P* < 0.01), relative to the non-irradiated controls (determined by Student t test).

**Figure 6:**
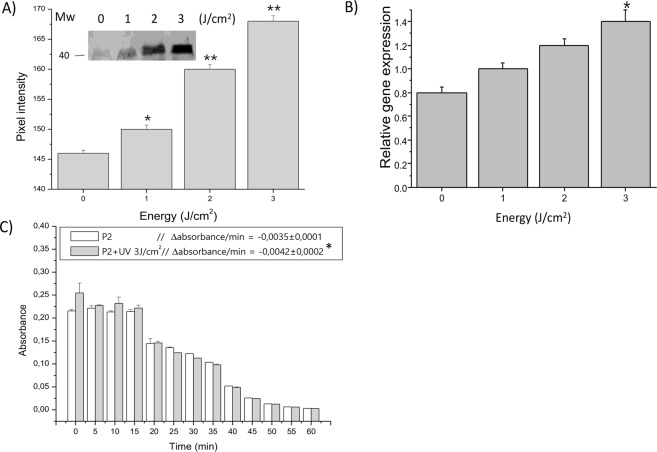
(A) Expression of Cu/Zn-SOD3 (CSD3) in *P*. *dicentrarchi* trophonts exposed to different levels of UV radiation, as determined in a Western blot (WB) assay with a polyclonal antibody against the recombinant protein rCSD3 (anti-rCSD3) and proteins in peak 2 (P2) purified by ion exchange chromatography. The graph corresponds to the densitometric analysis of the WB (n = 3). A representative WB (not cropped) is shown in the figure. (**B**) Expression of Cu/Zn-SOD3 transcripts by ciliates exposed to different doses of UV radiation and quantified by RT-qPCR. (**C**) SOD activity in the P2 fraction from ciliates treated by UV irradiation (3 J/cm^2^) and untreated ciliates (control), as determined by the PMS-NBT (phenazine methosulfonate - nitroblue tetrazolium) assay. The enzymatic activity was quantified as the decrease in absorbance at 560 nm/min (−Δabsorbance/min). The mean values and standard error are represented in the bar graph, and the asterisks indicate statistically significant differences (*P < 0.05; **P < 0.01) relative to the non-irradiated control.

Ciliates exposed to ultraviolet light generated a higher level of SOD isoenzymes than non-exposed ciliates (Fig. 5). The protein levels of CSD2-3, MSD and FSD increased significantly after exposure to ultraviolet radiation with an energy of 3 J/cm^2^. In this experiment, we also analyzed the possible existence of extracellular SODs secreted by the ciliate. To investigate this phenomenon, we use an ELISA to determine the SOD levels in the culture medium of irradiated and non-irradiated ciliates. The assay showed an increase in the amount of Pd-Cu/Zn-SOD2, suggesting that this isoenzyme is released extracellularly, and also in the amount of Pd-Cu/Zn-SOD3 (Fig. 5A,B). On the other hand, the Pd-Mn-SOD isoenzyme was not detected extracellularly (Fig. 5C), and the Pd-Fe-SOD isoenzyme was detected in the culture medium, although the levels of this enzyme decreased after irradiation of trophozoites (Fig. 5D). Expression of SOD enzymes, at both the protein level (Fig. 6A) and transcriptomic level (Fig. 6B), was dependent on the dose of irradiation administered. Finally, exposure of the ciliates to oxidative stress through the chemical generation of •O_2_^−^ in the culture medium caused a significant increase in SOD activity in the exposed trophozoites (Fig. 6C).

## Discussion

Eukaryotes possess three types of SOD families characterized by the presence of metal cofactors (Mn^2+^, Fe^3+^, Cu^2+^ or Zn^2+^) in the active sites, location in different organelles and cellular compartments, and different sensitivities to cyanide, azide and hydrogen peroxide^48,49^. In the staining of antioxidant activity on native polyacrylamide gels, three bands of activity corresponding to three SOD isoenzymes were observed in a soluble extract of *P*. *dicentrarchi* purified by anion exchange chromatography. Identification of the enzymatic activity bands of the SOD isoenzymes was initially based on sensitivity to several inhibitors. Thus, the activity of the Cu/Zn-SODs was identified by the sensitivity to NaCN^50^, while the Fe-SOD activity was identified by sensitivity to H_2_O_2_ and NaN_3_^51,52^. The Mn-SOD activity was identified because it was not inhibited by NaCN or by H_2_O_2_^53^. In this study, we also analyzed the effect of the phytoalexin trans-resveratrol (RESV), which has been shown to inhibit SOD in plants with an apparent K_i_ of 10 μM^54^. The inhibitory capacity of RESV on the SOD activity of *P*. *dicentrarchi* was also confirmed in a previous study, in which we found that a concentration of 100 μM RESV caused inhibition of SOD activity^55^. In the present study, we showed that RESV partly inhibited the activity of the Pd-Mn-SOD isoenzyme and completely inhibited the activity of the Pd-Fe-SOD isoenzyme, while the Pd-Cu/Zn-SODs isoenzymes were insensitive to this polyphenol.

With the purpose of identifying the protein sequences of the Pd-SOD family of enzymes, we carried out a transcriptomic analysis with an RNAseq assay to locate homologous sequences and to identify the molecules involved. Although it was initially believed that protozoa lacked genes that encode the Cu/Zn-SOD isoenzyme^56,57^, several studies have shown its existence and activity in these unicellular organisms, including, for example, the amphizoic ciliates and amoebas^24,58^. In this study, we detected the presence in *P*. *dicentrarchi* of three transcripts with different sequences and with showing some similarity to Cu/Zn-SOD enzymes. The existence of several Cu/ZnSOD forms is frequently observed among ciliates. Thus, in *Tetrahymena thermophila* three Cu/Zn-SOD genes have been identified that encode enzymes of MW 17.9–21.4 kDa; in *Euplotes focardii* two genes that encode proteins of MW 16.8–20 kDa; and in *Oxytrichia trifallax* two genes that encode proteins of MW 17.5–17.6 kDa^24^. In addition, two types of Cu/Zn-SOD (Ec-Cu/Zn-SOD1 and EC-Cu/Zn-SOD2) identified in *E*. *crassus* express proteins of sizes between 17.3 and 19.9 kDa and pI of 4.98 and 6.65, respectively; the enzyme corresponding to type 1 has a signal peptide for extracellular activity^25^. In this study, the CSD2 protein encoded by the Pd-Cu/Zn-SOD2 transcript also has a signal peptide that indicates a relationship with a function in extracellular activity, while the CSD3 protein encoded by the Pd-Cu/ZnSOD3 transcript has a transmembrane region, indicating that it is attached to membranes. In the nematode *Caenorhabditis elegans*, the presence of Cu/Zn-SOD isoforms with a consensus signal peptide at the N-terminus and similar to the extracellular-types of Cu/Zn-SODs in mammals, has also been detected and associated with membranes with a presumed transmembrane domain at the C-terminal region generated through an alternative splicing process^59^. Other parasites such as the helminth *Schistosoma mansoni* also have extracellular Cu/Zn-SODs forms that contain a signal peptide and membrane-associated transmembrane regions^60^. Likewise, the presence of four variants in the isoelectric point (pI) has also been observed in adult forms of the same parasite, although in all cases the pI of the Cu/Zn-SODs was slightly lower than 7^60^. In the Pd-Cu/Zn-SODs transcripts, the pI of a transcript that encodes the protein CSD1 is below 7 and in two transcripts that encode proteins CSD2 and CSD3, the value of pI is greater than 7, indicating that pI is lower than 7 in cytoplasmic forms, while it is greater than 7 in extracellular forms or form associated with membranes. The existence of extracellular forms with pI > 7 has also been observed in plants, and the extracellular forms of the Cu/Zn-SODs have a higher pI than cytosolic forms^61,62^. Modelling of the proteins encoded by the three transcripts associated with Pd-Cu/Zn-SODs indicates that these are oligomeric proteins of the homodimer type, which may indicate that the MW of the active enzymes in their native form will be between 34 and 44 kDa. In most eukaryote organisms, Cu/Zn-SODs are presented as homodimer enzymes^49,63^. In the marine ciliate *E*. *focardii* and the amoeba *Acanthamoeba castellani*, the enzymes Cu/ZnSODs are homodimeric^26,58^. The low homology between the AA sequences of the Cu/Zn-SOD in *P*. *dicentrarchi* seems to indicate that these transcripts are derived from the expression of paralogous genes, as occurs in some archaebacteria^64^.

The Fe- and Mn-SOD enzymes are the oldest SOD group and have probably evolved from orthologous genes^20^. Fe-SOD enzymes are found in prokaryotes and in chloroplasts, while Mn-SODs occur both in prokaryotes and in the mitochondrial matrix of eukaryotes^26^. It has been suggested that iron may have been the first metal used as a cofactor associated with the active site of the first SOD due to its abundance at that time in the form of soluble Fe^2+^^65^. In protozoa, the Mn-SOD isoenzyme has been detected in *Euglena gracilis*^66^ and in the ciliates *Euplotes focardii* and *E*. *crassus*^25,26^. Fe-SOD was originally considered a bacterial cytosolic enzyme; however, it has also been detected in archaea, in plant chloroplasts, as well as in the cytosol, glycosomes and mitochondria of protists^67^. Fe-SODs are common in Protozoa, e.g. in the amitochondriate *Entamoeba histolytica*^68^, as well as in *T*. *pyriformis*^69^, *Plasmodium falciparum*^70^, *Leishmania chagasi*^71^, *Trypanosoma cruzi*^72^, *Perkinsus marinus*^73,74^ *and Trichomonas vaginalis*^75^. The Fe-SOD form seems to predominate in anaerobic or microaerophilic organisms, while the Mn-SOD form predominates in aerobes^53^. The presence of an Fe-SOD form in *P*. *dicentrarchi* may be an adaptation to its microaerophilic nature and sensitivity to high concentrations of oxygen because it is a benthic organism^76^. The Pd-Mn-SOD isoenzyme possesses four Mn binding sites, as do other Mn-SODs of eukaryotes^77^. In bacteria and eukaryotes, Fe-and Mn-SOD exist in both the homodimeric and homotetrameric forms with 22 kDa subunits^67,77^; however, in *P*. *dicentrarchi*, as in the symbiotic dinoflagellate protozoon *Symbiodinium* or the apicomplex *P*. *falciparum*, both Mn-SOD and Fe-SOD represent dimeric forms^78,79^. By contrast, the Fe-SOD isolated from the ciliate *T*. *pyriformis* is reported to be tetrameric^69^. In parasitic protists such as *T*. *vaginalis*, Fe-SOD is a dimeric protein that displays high structural similarity to Fe-SODs of prokaryotes, possibly indicating that its presence in eukaryotes may be due to an endosymbiotic process^80^. The CSD3 protein encoded by Pd-Cu/Zn-SOD3 transcript and the FSD protein encoded by Pd-FeSOD transcript are both phylogenetically related to ciliated SODs and these proteins show greater identity with SODs of the scuticociliate *P*. *persalinus*^81^.

The bimetallic enzymes copper-and zinc-containing SODs are a family of isoenzymes found in both intracellular and extracellular locations. This family comprises ubiquitous enzymes that appear primarily in the cytosol but can also occur in the mitochondrial intermembrane space, the secretory pathway and even the nucleus^82^. In the present study, the bioinformatic prediction and the immunofluorescence findings indicate that the Pd-Cu/Zn-SOD3 form is in the alveolar sacs, in a similar way that the Cu/Zn-SODs of bacteria occur in the periplasm^83^. The other two Pd-Cu/Zn-SODs appear to occur in the cytosol (Pd-Cu/Zn-SOD1) and extracellularly (Pd-Cu/Zn-SOD2). In eukaryotes, the presence of cytosolic and extracellular Cu/Zn-SODs is very common^84,85^. The presence of a signal peptide in Pd-Cu/ZnSOD2 seems to indicate that this enzyme is extracellular. As •O_2_^−^ is generally unable to cross the cell membrane, the substrate for this Cu/Zn-SOD in eukaryotes should be produced outside the cell^82^.

The identity analysis of the 220 AA transcript, determined using the Blastp tool, indicates that it possesses the highest identity with a SOD [Fe] protein in *T*. *thermophila* SB210 and with an Mn/Fe-SOD in the scuticociliate *P*. *persalinus*^81^. Fe- and Mn-SODs are found in a wide variety of species and may be located either in the cytosol or in the mitochondria or in both; however, in animals Mn-SOD is usually present in the mitochondria^57^. The Mn-SOD enzyme in eukaryotes is synthesized in the cytosol and exported post-translationally to the mitochondrial matrix where 90% of cellular O_2_ is consumed^86^. The results of the immunofluorescence assay indicate that the protein encoded by the 220 AA transcript occurs in the mitochondria. In *P*. *dicentrarchi*, the protein encoded by the 220 AA transcripts also sends an export signal to the mitochondria and potentially has a homodimeric structure. Together with its predicted molecular size and its insensitivity to inhibition by H_2_O_2_ and NaN_3_, the above findings seem to indicate that the protein is an Mn-SOD with mitochondrial localization. With respect to the transcript generated by the 249 AA protein, Blastp analysis of the NCBI indicates that the maximum identity of this sequence occurs with bacterial SOD sequences. However, use of this tool to perform the search in the database of *Tetrahymena* yielded maximum identity with an Fe-SOD of *T*. *borealis* and with an Mn/Fe-SOD of *Oxitrichia*. These results are also consistent with the prediction of the InterPro bioinformatics program, which indicates that this protein has a signal peptide, lacks transmembrane regions and possesses domains of the Mn/Fe-SODs family. In addition, considering the molecular size of this enzyme in its homodimeric form, the inability of cyanide to inhibit its enzymatic activity and its sensitivity to azide together indicate that this enzyme contains iron as a metallic cofactor in the active site^80^.

In the marine environment where the free-living cilia live, various environmental changes can lead to the generation of a high level of oxidative stress^18,26^. During infection, the ciliates are also subjected to high levels of ROS generated by the cells of the host’s innate immune system^87^. Among the factors that can intervene the following are environmentally most important in generating high levels of ROS: temperature, excess oxygen in water, solar UV radiation and the presence of various types of pollutants^18,88^. Likewise, when fish are cultured in open-circuit farms, the water is commonly oxygenated by the direct supply of O_2_ or by aeration, and disinfection is carried out by UV radiation^89,90^. In order to survive, marine organisms use SOD enzymes that allow them to eliminate not only the endogenous •O_2_^−^ produced during metabolic processes, including mitochondrial respiration, but also the exogenous •O_2_^−^ present in environments with high level of oxidative stress^57^. In the present study, we observed that the levels of expression of all SODs increase significantly after exposure of trophonts to UV radiation. Likewise, we also observed that both Pd-Cu/Zn-SOD2 and Pd-Fe-SOD are excreted into the culture medium after UV irradiation. These findings indicate the existence of extracellular forms of Pd-Cu/Zn-SOD and of Pd-Fe-SOD. In both cases, although the cellular localization was initially predicted to be cytosolic, the existence of signal peptides in both isoenzymes seems to confirm that these proteins can be secreted and participate in the neutralization of extracellular •O_2_^−^ generated by UV radiation. Mammals also possess a Cu/Zn-SOD and an extracellular Fe-SOD induced under oxidative stress^91^. Cytosolic and extracellular Cu/Zn-SODs have also been identified in several parasitic organisms^92–94^. In the marine ciliate *E*. *focardii*, one of the Cu/Zn-SODs is extracellular^26^. An excreted Fe-SOD has also been detected in the Tripanosomatid belonging to the genus *Phytomonas*, and which also has an immunogenic capacity^22^. Expression of the Pd-Cu/Zn-SODs, at both the protein and transcription levels increase in proportion to the dose of UV radiation administered, and the enzymatic activity is also significantly increased at the highest dose of non-lethal UV radiation.

In conclusion, *P*. *dicentrarchi* possesses the three characteristic types of SODs present in eukaryotes, i.e. Cu/Zn-SOD, Mn-SOD and Fe-SOD, the functional forms of which are oligomeric enzymes of the homodimeric type. Three types of Pd-Cu/Zn-SOD enzymes are sensitive to NaCN: one is cytosolic, another is present in the alveolar sacs of the trophonts and a third is extracellular. All three have very different aa sequences. Another two other isoenzymes have also been identified: a mitochondrial Mn-SOD insensitive to H_2_O_2_ and NaN_3_, and an extracellular cytosolic Fe-SOD sensitive to H_2_O_2_ and NaN_3_. The activity of all SODs isoenzymes increases under conditions of oxidative stress induced by UV radiation. This study highlights the role of SODs as enzymes that protect the ciliate from the toxic action of •O_2_^−^ generated both in the marine environment, during its free life phase, and in the host, during the parasitic phase. The extracellular forms of these enzymes, with very different aa sequences from the host, may yield potential diagnostic targets, as well as potential antigens, in order to produce vaccines in the future^95,96^.

## Data Availability

The datasets generated during and/or analysed during the current study are available from the corresponding author on reasonable request.
